# Concordance of End-of-Life Care With End-of-Life Wishes in an Integrated Health Care System

**DOI:** 10.1001/jamanetworkopen.2021.3053

**Published:** 2021-04-06

**Authors:** David P. Glass, Susan E. Wang, Paul M. Minardi, Michael H. Kanter

**Affiliations:** 1 Department of Research and Evaluation, Kaiser Permanente Southern California; 2 Department of Health Systems Science, Bernard J. Tyson School of Medicine, Kaiser Permanente, Pasadena, California; 3 Life Care Planning and Serious Illness Care, Southern California Permanente Medical Group, Los Angeles; 4 Washington Permanente Medical Group, Renton; 5 Department of Clinical Science, Bernard J. Tyson School of Medicine, Kaiser Permanente, Pasadena, California

## Abstract

**Importance:**

There is widespread consensus on the challenges to meeting the end-of-life wishes of decedents in the US. However, there is broad but not always recognized success in meeting wishes among decedents 65 years and older.

**Objective:**

To assess how well end-of-life wishes of decedents 65 years and older are met in the last year of life.

**Design, Setting, and Participants:**

This quality improvement study involved 3 planned samples of family members or informants identified as the primary contact in the medical record of Kaiser Permanente Southern California decedents. The first sample was 715 decedents, 65 years or older, who died between April 1 and May 31, 2017. The second was a high-cost sample of 332 decedents, 65 years or older, who died between June 1, 2016, and May 31, 2017, and whose costs in the last year of life were in the top 10% of the costs of all decedents. The third was a lower-cost sample with 655 decedents whose costs were not in the top 10%. The survey was fielded between December 19, 2017, and February 8, 2018.

**Main Outcomes and Measures:**

Meeting end-of-life wishes, discussions with next of kin and physicians, types of discordant care, and perceptions of amount of care received.

**Results:**

Surveys were completed by 715 of the 2281 next of kin in the all-decedent sample (mean [SD] decedent age, 80.9 [8.9] years; 361 [50.5%] male) for a 31% response rate; in 332 of the 1339 next of kin in the high-cost sample (mean [SD] decedent age, 75.5 [7.1] years; 194 [48.4%] male) for a 25% response rate; and in 659 of 2058 in the lower-cost sample (mean [SD] decedent age, 81.6 [8.8] years) for a 32% response rate. Respondents noted that high percentages of decedents received treatment that was concordant with their desires: 601 (88.9%) had their wishes met, 39 (5.9%) received a treatment they did not want, and 554 (84.1%) filled out an advance directive. A total of 509 respondents (82.5%) believed the amount of care was the right amount. Those with the highest costs had their wishes met at lower rates than those with lower costs (250 [80.1%] vs 553 [89.6%]).

**Conclusions and Relevance:**

In this Kaiser Permanente Southern California cohort, a large proportion of decedents 65 years and older had end-of-life discussions and documentation, had their wishes met, and received the amount of care they thought appropriate.

## Key Points

**Question:**

How well are the end-of-life wishes of decedents 65 years or older met?

**Findings:**

In this quality improvement study of next of kin of 715 decedents who died at 65 years or older, respondents reported that 80% to 90% of decedents discussed preferences with next of kin, filled out an advance directive, or gave real thought to the care they desired. Respondents also reported that 85% to 95% of decedents gave positive responses to receiving the care they wanted and 82.5% of decedents believed they received the right amount of care.

**Meaning:**

This study suggests that most older decedents are receiving care concordant with their wishes.

## Teaser

This quality improvement study assesses how well end-of-life wishes of decedents 65 years and older in a large US health care system are met in the last year of life.

## Introduction

The research on end-of-life care in the US is extensive, deep, and long-standing. Reflecting the interest in and concern with this area, the Institute of Medicine (IOM) has produced 2 substantial reports on the situation in the US and recommendations for improvement.^1,2^ At the heart of those reports is the recognition that there is still a long road ahead to the achievement of patient-centered care.

There is widespread consensus on 3 challenges in end-of-life care in the US. The first has to do with the lack of discussion and documentation of patients’ preferences regarding end-of-life care.^3,4,5,6,7,8,9^ Fears about death and the failure to discuss or document end-of-life preferences are common in the US.^10,11,12^ The lack of knowledge of patient preferences obviously makes it difficult to match appropriate treatments with patient priorities.

The second, related concern is that patients’ wishes are often not met.^6,13,14^ Patients end up in situations in which they receive discordant care.^15,16^ Physicians provide care that is neither wanted nor needed because, lacking information on preferences, they default to providing all possible care. Patients conversely may want certain care but not receive it because of poor communication with their physician.^17^ Discordant care has many dimensions, including the location of death, the treatments received, the pain endured, and the quality of life experienced.^18,19,20,21^

A third shortcoming to serious illness care is that patients receive far more care than desired and that health care costs are, as a result, unnecessarily high.^22,23,24,25^ The large portion of Medicare costs spent during the last year of life (approximately 25%) coupled with the variability of these costs and treatments across geographic areas has convinced many that end-of-life spending is an area of waste and large potential savings.^26,27,28,29^

A survey was conducted as a baseline to understand how well our medical system was delivering end-of-life care. We were interested in measuring our performance on each of these 3 challenges with the longer-term goal of identifying where and how we might improve our end-of-life care. We gathered information from next of kin about their loved ones’ experiences in the last year of life. In alignment with the conceptual model of achieving goal-concordant care,^30^ we wished to know the degree to which loved ones had communicated their end-of-life preferences, how well those wishes had been met, and whether loved ones received more care than desired. The first objective was to understand our gaps in these areas. We also explored, as secondary objectives, the association of costs in the last year of life with concordance and the association of different end-of-life values (wanting all end-of-life health care measures used vs sometimes allowing patients to die) with meeting end-of-life wishes. We hypothesized that both those with higher costs and those who wanted all end-of-life health care measures used would have lower concordance, reflecting the challenges noted above. However, our exploratory findings did not neatly match these challenges and hypotheses, offering a different perspective on the current situation and the path toward improvement.

## Methods

This survey questionnaire and protocol were reviewed by the Kaiser Permanente Southern California (KPSC) Institutional Review Board, which determined that this was not human participant research, did not require consent, and was a quality improvement project. All data were deidentified. Quality improvement is designed to provide immediate health delivery improvements in a particular setting.^31,32^ This study followed the American Association for Public Opinion Research (AAPOR) reporting guideline.

### Setting

This survey was conducted at KPSC, which is a large integrated health care system that provides insurance and medical services to more than 4.6 million members at 15 hospitals and 231 medical offices scattered throughout Southern California. The member population of KPSC is demographically diverse and broadly representative of groups living in Southern California.^33^

### Participants

This survey was conducted with the next of kin of deceased members in KPSC who were 65 years or older, had 2 or more visits in the last year of their life, and died between April 1 and May 31, 2017 (all-decedent sample). The next of kin was the primary contact in the decedent’s health record. The survey was fielded between December 19, 2017, and February 8, 2018.

A second, high-cost sample of deceased members was also drawn. These were deceased members, 65 years or older, with 2 or more visits in the last year of their life, who died between June 1, 2016, through May 31, 2017 (to obtain sufficient sample), and whose costs during the last year of life were in the top 10% of the costs of all members who died during this period.

A third sample, deemed lower-cost decedents, was created from the first sample described above as a comparison group to the high-cost sample (eAppendix 1 in the Supplement). These were decedents whose costs in the last year of life were not in the top 10% in costs.

All of the results are reported by next of kin on behalf of their loved ones. We sometimes refer to decedents’ values and beliefs, but this is as perceived and reported by their next of kin.

### Questionnaire Development

A 4-step process (eAppendix 2 in the Supplement) was used in developing the questionnaire: (1) existing end-of-life questionnaires were reviewed, and questions that focused on treatment concordance were adopted^5,12,34,35,36,37,38,39,40,41,42^; (2) key questions were tested in focus groups with next of kin; (3) a draft questionnaire was reviewed by internal KPSC end-of-life experts; and (4) a pretest of the questionnaire with next of kin was conducted. The questionnaire is provided in eAppendix 3 in the Supplement and covers the 4 domains of end-of-life communications: preferences and values, treatments and care received, and concordance.^30^ Item nonresponse for key measures was small (<2.0%).

### Study Procedures

Two mailings of the questionnaire were sent to the address of the primary contact when available (approximately 35% of the time) or otherwise to the decedent’s address. If no response was received, up to a maximum of 10 telephone calls were made to next of kin. However, 85% of the completions were by mail (involving no telephone calls) and 15% were completed by telephone. The mean number of contacts was 2.1 per respondent. Additional survey procedure information in provided in eAppendix 4 in the Supplement.

The total costs in the last year of life of all those who died between June 1, 2016, through May 31, 2017, were obtained from the Heath Plan Accounting Office at KPSC. These costs included all major medical costs both inside and outside the KPSC system (eAppendix 5 in the Supplement).

Nonresponse weights were developed for all samples using logistic regression.^43^ Included factors were sex, race/ethnicity, age, and costs during the last year of life. A comparison of nonrespondents and respondents can be found in eAppendix 6 in the Supplement.

### Statistical Analysis

The characteristics of the respondents were described for the study samples. Categorical variables were described as numbers (percentages). A Pearson χ^2^ test was used to examine differences between groups. We considered a 2-tailed *P* ≤ .05 as statistically significant. Statistical analyses were performed with SAS software, version 9.4 (SAS Institute Inc) and SPSS software, version 20 (IBM Corp).

Differences in the measures were examined for those who had higher costs (top 10%) vs lower costs (bottom 90%) in the last year of life as well as for those who reported views closer to the statement “In all circumstances, doctors and nurses should do everything possible to save the life of a patient” vs “Sometimes there are circumstances where a patient should be allowed to die.”

An ordinary least squares regression was run to identify significant independent variables and their association with the overall concordance variable (agreement with the statement “Kaiser Permanente gave care and treatment over the last year of my loved one’s life that met my loved one’s wishes.”). The regression model was then used to ascertain the estimated change in this dependent variable if the performance on each independent variable was fixed (eg, association if all decedents had had end-of-life discussions). Because the model is linear, this is equivalent to an estimated margin calculation for the maximum improvement.^44^ Details about the regression and the estimated change are in eAppendix 7 in the Supplement.

## Results

Surveys were completed in the all-decedent sample (mean [SD] decedent age, 80.9 [8.9] years; 361 [50.5%] male), with 715 of the 2281 next of kin invited to participate for a 31% response rate. Surveys were completed in the high-cost sample (mean [SD] decedent age, 75.5 [7.1] years; 194 [48.4%] male), with 332 of the 1339 next of kin invited to participate, for a 25% response rate. Surveys were completed in the lower-cost sample (mean [SD] decedent age, 81.6 [8.8] years; 328 [50.1] female), with 659 of the 2058 next of kin invited to participate, for a 32% response rate.

### Characteristics of the Samples 

Table 1 gives the characteristics of the 3 samples in this study. In the all-decedent sample, the reported rates of receiving specific treatments in the last year of life were 13.7% (n = 91) for cardiopulmonary resuscitation, 18.7% (n = 125) for mechanical respiration, and 10.5% (n = 71) for artificial feeding. The findings in the higher-cost sample were significantly different from those in the lower-cost sample, with 72 (26.2%) in the higher-cost samples vs 81 (13.4%) in the lower-cost sample receiving cardiopulmonary resuscitation, 149 (48.5%) in the higher-cost sample vs 100 (16.2%) in the lower-cost sample receiving mechanical respiration, and 117 (38.6%) in the higher-cost sample vs 51 (8.3%) in the lower-cost sample receiving artificial feeding. The higher-cost sample incurred nearly 5 times the costs of the lower-cost sample in the last year of life.

**Table 1.** Characteristics of the 3 Samples in the End-of-Life Concordance Study

Characteristic	Decedent sample^a^			*P* value for higher vs lower cost^b^
	All (N = 715)	Higher cost (top 10% of costs) (n = 332)	Lower cost (bottom 90% of costs) (n = 655)	
Sex				
Female	354 (49.5)	138 (41.6)	328 (50.1)	.01
Male	361 (50.5)	194 (58.4)	326 (49.9)	
Age, y				
65-74	207 (28.9)	171 (51.5)	167 (25.6)	<.001
75-84	239 (33.4)	119 (35.9)	223 (34.0)	
≥85	270 (37.8)	42 (12.5)	265 (40.4)	
Age, mean (SD), y	80.9 (8.9)	75.5 (7.1)	81.6 (8.8)	
Race/ethnicity				
Hispanic	138 (19.3)	65 (21.2)	122 (19.7)	.007
Non-Hispanic				
White	388 (54.2)	146 (47.2)	361 (58.0)	
Black	88 (12.3)	54 (17.6)	85 (13.6)	
Other^c^ or unknown	101 (14.1)	43 (14.0)	54 (8.7)	
Educational level				
High school or lower	276 (38.6)	101 (31.1)	261 (40.6)	.007
Some college (AA or AS)	195 (27.3)	103 (31.5)	173 (27.0)	
4-Year college (BA or BS)	121 (16.9)	62 (19.2)	109 (17.0)	
Graduate or professional degree	90 (12.6)	56 (17.3)	82 (12.7)	
Unknown	33 (4.6)	3 (0.9)	18 (2.8)	
Treatments received in last year of life				
CPR	91 (13.7)	72 (26.2)	81 (13.4)	<.001
Mechanical respiration	125 (18.7)	149 (48.5)	100 (16.2)	<.001
Artificial feeding	71 (10.5)	117 (38.6)	51 (8.3)	<.001
Costs in last year of life, % of mean costs in last year in all decedent sample	100	351	74	NA

Abbreviations: AA, associate of arts; AS, associate of sciences; BA, bachelor of arts; BS, bachelor of science; CPR, cardiopulmonary resuscitation; NA, not applicable.

^a^ Data are presented as number (percentage) of decedents unless otherwise indicated.

^b^ Significance is from Pearson χ^2^ test.

^c^ Other included Asian, American Indian/Alaska Native, Hawaiian/Other Pacific Islander, and Other.

### End-of-Life Discussion, Knowledge, and Thought Given to Treatment Preferences

Table 2 presents the communication exchanges of the all-decedent sample, indicating that most decedents had engaged in these activities before dying. A total of 579 decedents (82.6%) had had discussions with their next of kin, and the next of kin professed high levels of knowledge (557 [79.7%]) about preferences and familiarity with health care decisions (648 [91.2%]). A total of 554 decedents (84.1%) had completed an advance directive. A total of 291 decedents (55.4%) reported having had an in-depth discussion and 51 (9.7%) said they had some discussion about end-of-life preferences.

**Table 2.** End-of-Life Discussions, Knowledge, and Thought Given to Treatment Preferences in the All-Decedent Sample

Measure	Decedents, No. (%) [95% CI]
Had discussion with next of kin about end-of-life care and treatment preferences	
Yes	579 (82.6) [79.8-85.4]
No	122 (17.4) [14.6-20.2]
Next-of-kin knowledge about loved one’s end-of-life care and treatment preferences	
A lot	557 (79.7) [76.7-82.7]
Some	99 (14.2) [11.6-16.8]
Not too much	35 (5.0) [3.4-6.6]
Nothing at all	8 (1.1) [0.4-1.9]
Next-of-kin familiarity with loved one’s health care decisions in the last year of life	
Very familiar	648 (91.2) [89.2-93.3]
Somewhat familiar	45 (6.3) [4.5-8.1]
Not too familiar	11 (1.5) [0.6-2.4]
Not at all familiar	7 (0.9) [0.2-1.6]
Loved one had a discussion with Kaiser Permanente physician or staff about end-of-life treatment preferences	
Yes	
In-depth discussion	291 (55.4) [51.2-59.7]
Regular discussion	51 (9.7) [7.1-12.2]
No	183 (34.9) [30.8-39.0]
Loved one had filled out an advance directive	
Yes	554 (84.1) [81.3-86.9]
No	105 (15.9) [13.1-18.7]
Level of thought loved one had given to end-of-life treatments wanted or not wanted	
Given real thought	491 (78.0) [74.8-81.3]
Not much thought	138 (22.0) [18.8-25.2]

### General Measures of Concordance

By and large, decedents received care that met their wishes on several questions focused on this issue (Table 3). For example, among the all-decedents sample, 601 (88.9%) agreed that their loved one’s care and treatment wishes were met in the last year of life. Similarly, 39 (5.9%) reported receiving an unwanted treatment, and 84 (13.5%) reported not receiving a desired treatment. We also asked about the amount of care received in the last year of life, and 509 (82.5%) reported that they were given the right amount of care. We compared concordance between those who wanted every lifesaving treatment vs allowing patients to die and found no differences on 5 of the 6 measures. On the other hand, the higher-cost group (top 10%) was less satisfied than the lower-cost group with their care and treatment on all but one of the measures.

**Table 3.** General Measures of Concordance

Measure	All-decedent sample, No. (%) (N = 715)	All end-of-life health care measures used, No. (%) (n = 152 [25%])	Allow to die group (n = 455 [75%])	*P* value (all end-of-life health care measures used vs allow to die)^a^	Top 10% in cost sample, No. (%) (n = 332)	Bottom 90% in cost sample, No. (%) (n = 655)	*P* value (top vs bottom cost)^a^
Met wishes							
Strongly agree or agree	601 (88.9)	122 (85.9)	403 (91.8)	.06	247 (79.8)	551 (89.5)	<.001
Strongly disagree or disagree	75 (11.1)	20 (14.1)	36 (8.2)		63 (20.2)	65 (10.5)	
Satisfaction with way died							
Very satisfied or satisfied	133 (85.4)	13 (72.2)	110 (90.9)	.16	54 (70.2)	123 (85.3)	.028
Very dissatisfied or dissatisfied	23 (14.6)	5 (27.8)	11 (9.1)		23 (29.8)	21 (14.7)	
Received treatment not wanted							
Yes	39 (5.9)	7 (5.3)	23 (5.4)	.98	24 (8.8)	36 (5.9)	.129
No	619 (94.1)	125 (94.7)	405 (94.6)		252 (91.2)	572 (94.1)	
Not able to get desired treatment							
Yes	84 (13.5)	19 (15.4)	54 (13.0)	.495	58 (21.2)	75 (13.1)	.003
No	535 (86.5)	104 (84.6)	360 (87.0)		217 (78.8)	493 (86.9)	
Amount of care							
Too much	21 (3.4)	2 (1.6)	17 (4.2)	.007	9 (3.4)	21 (3.6)	<.001
Right amount	509 (82.5)	99 (77.3)	346 (84.8)		183 (69.2)	473 (83.4)	
Too little	87 (14.1)	27 (21.1)	45 (11.0)		72 (27.4)	74 (13.0)	
Rating of overall care in last month							
Excellent, very good, or good	579 (87.7)	117 (83.6)	389 (90.3)	.33	253 (79.9)	529 (87.8)	<.001
Fair/poor	81 (12.3)	23 (16.4)	42 (9.7)		64 (20.1)	73 (12.2)	

^a^ Significance is from Pearson χ^2^ test.

### Types and Levels of Discordant Care

Although the results on general measures of concordance are high, there are specific areas where concordance remains a challenge. Three such areas are presented in Table 4. First, only 338 (57.1%) of those who said they would like to die at home did so. Second, few decedents received cardiopulmonary resuscitation, mechanical respiration, or artificial feeding who did not want it. However, among only those who received these treatments, the rates of discordance were substantial. For example, 28 (37.9%) of those who received cardiopulmonary resuscitation reported that they did not want it, with similar findings for mechanical respiration (44 [42.7%]) and artificial feeding (20 [39.1%]). Finally, we asked how often decedents’ pain made them uncomfortable. A total of 182 (28.4%) reported they were always in pain, and 147 (22.8%) reported that they were usually in pain. The levels of pain reported by those in hospice and/or palliative care were not much different (eAppendix 8 in the Supplement).

**Table 4.** Types and Levels of Discordant Care in the All-Decedent Sample

Measure	Decedents, No. (%) [95% CI]
Location of death among those wishing to die at home^a^	
Home	338 (57.1) [53.1-61.1]
Hospital	180 (30.3) [26.6-34.0]
Nursing home or skilled nursing facility	27 (4.6) [2.9-6.3]
Hospice facility	18 (3.0) [1.6-4.4]
Assisted living facility	15 (2.6) [1.3-3.9]
Somewhere else	14 (2.4) [1.2-3.6]
Received treatment they did not want	
CPR^b^	28 (4.8) [3.0-6.5]
Mechanical respiration^b^	44 (7.3) [5.2-9.4]
Artificial feeding^b^	20 (3.4) [1.9-4.9]
Received treatment they did not want, among those who received that treatment	
CPR^c^	28 (37.9) [26.5-49.2]
Mechanical respiration^d^	44 (42.7) [32.9-52.4]
Artificial feeding^e^	20 (39.1) [25.3-52.8]
How often loved one’s pain made him/her uncomfortable	
Always	182 (28.4) [24.9-31.9]
Usually	147 (22.8) [19.6-26.1]
Sometimes	219 (34.1) [30.4-37.8]
Never/did not have any pain	94 (14.7) [12.0-17.5]

Abbreviation: CPR, cardiopulmonary resuscitation.

^a^ A total of 88% of the sample wished to die at home.

^b^ The total numbers on whom these percentages are based are as follows: CPR,  589; mechanical respiration, 602; and artificial feeding, 596.

^c^ A total of 14% of the sample received CPR (n = 74).

^d^ A total of 19% of the sample received mechanical respiration (n = 103).

^e^ A total of 11% of the sample received artificial feeding (n = 52).

### Key Factors and Their Association With Meeting Wishes

Several factors appear promising for improving the percentage of respondents strongly agreeing that their wishes were met. The Figure provides a ranking of the 6 attributes significantly associated with wishes met. The Figure shows by how much strong agreement that wishes were met would increase if that attribute was met for everyone. Two attributes stand out as the most promising. The first is trying to accommodate decedents’ desired place to die. The results indicate that if all decedents believed that physicians and staff had done a great deal to accommodate their wishes concerning location, the percentage strongly agreeing their wishes were met would increase by 11.4 percentage points. The second most important attribute is end-of-life discussions. If all decedents had discussions about end-of-life preferences with a physician or other medical staff member, the percentage strongly agreeing their wishes were met would increase by 9.8 percentage points.

**Figure.**
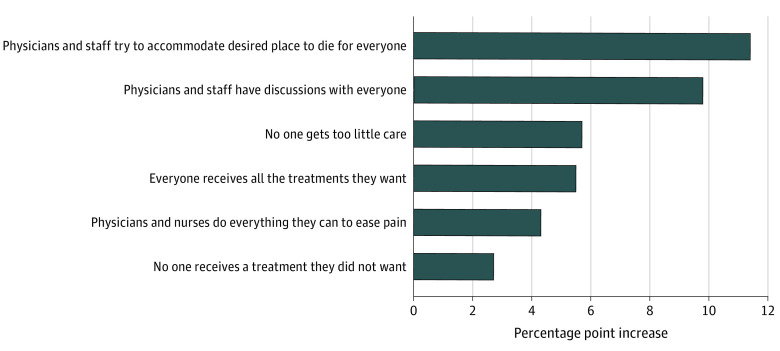
Expected Increase in Wishes Met if 100% of Decedents Receive Key Interventions Data are the expected percentage point increase in those who strongly agree with the statement “Kaiser Permanente gave care and treatment over the last year of my loved one’s life that met my loved one’s wishes.” The 6 items listed are the significant drivers from an all-decedent sample found in an ordinary least squares regression (*R*^2^ = .43) associated with meeting end-of-life wishes.

## Discussion

This quality improvement study found positive results in the 3 areas often described as challenges. First, the study found that approximately 80% to 90% of decedents discussed preferences with next of kin, filled out an advance directive, or gave real thought to the care they desired. Moreover, 65.0% had discussed their treatment preferences with physicians or other medical staff members. Second, the study found that approximately 85% to 95% of decedents gave positive scores on receiving the care they wanted. Third, most decedents thought they got the right amount of care (82.5%).

Beyond these core findings, 2 additional results are striking. One is that high rates of meeting end-of-life wishes extended to those holding different values. Those who wanted all end-of-life health care measures used and those who preferred less intrusive care both reported that their wishes were met. Second, those who had the highest level of costs in the last year of life were less satisfied on many concordance measures than those with lower costs.

Why does this generally positive picture of end-of-life care emerge among KPSC decedents? A careful review of the literature suggests that 2 factors may be involved. One is the age of the population studied. Many studies^4,6,12,45^ do not limit their focus to those 65 years or older but rather look at adults 18 years or older or an age cohort much younger than 65 years. Moreover, many studies^5,46^ do not focus on those who have died. Given that the goal of end-of-life planning is to optimize care at the end of life, studying patients who do not die is a biased approach. It is intuitive that the various elements of advance care planning will have more relevance and be more readily adopted by older individuals who ultimately died.

The 2014 IOM study^2^ provides backing for this. The IOM report examines 3 surveys and notes that the percentage of adults reporting to have an advance directive varies from 23% (in a study of adults ≥18 years of age) to 47% (in a study of adults ≥40 years of age) to 54% (in a study of adults ≥60 of age). None of these finding reach the 84.1% of decedents (≥65 of age) in the current study with an advance directive, but the gap lessens considerably. Moreover, the current data also support this same pattern, with advance directive completions increasing from 75.7% (65-74 years of age) to 82.2% (75-84 years of age) to 92.4% (≥85 years of age). The IOM study^2^ reports a similar pattern among age groups in holding discussions as does a California Health Care Foundation study^5^ (eAppendix 9 in the Supplement).

One consequence of this lack of standard definitions and approaches is that the results of this study tend to align with those studies that have used similar methods and diverge from studies that took different approaches. For example, on an overall measure of concordance, 3 studies relied on next-of-kin reports for decedents 65 years and older and reported that the percentages of patients whose wishes met were 87.4%,^47^ 81.1%,^20^ and 76%^48^—not so different from the 88.9% found in the current study. However, a study^49^ of patients with advanced cancer found only 68% received care consistent with preferences. A California next-of-kin survey^6^ reported that only 33% thought the end went the way their loved one wanted it to go. Finally, a methodological review of many studies found, “Proportions of patients who received concordant care varied from 14% to 98%.”^50^^(p 491)^The positive results of the current study, then, may understandably strike some as novel and others as confirmatory.

This focused study of those 65 years and older who died led to at least 2 novel findings beyond those already mentioned. One is why the high cost group was less satisfied. The study found that this high-cost group died at a substantially younger age than the lower-cost group, with half of the high-cost group dying between the ages of 65 and 74 years vs only one-quarter of the lower-cost group (Table 1). It seems likely that the high-cost group is high cost, at least in part, because the patients, families, and physicians all expected a life span beyond that and tried hard to extend it. The dissatisfaction of the high-cost group may well reflect the disappointment that despite all the efforts and costs, death came earlier than expected.

Another novel finding relates to the 2014 IOM report, which states, “In the end-of-life arena, there are opportunities for savings by avoiding acute care services that patients and families do not want and that are unlikely to benefit them.”^2^^(p 15)^ Although some savings opportunities may exist, this study finds little support that patients and families will lead this charge. Most decedents (82.5%) believed that they received the right amount of care, and the next largest group (14.1%) thought they received too little care. The definition of too much care in the last year of life, where to find it, and how to reduce it are all complicated issues.^26,51,52,53^ However, patients and families do not see this. Their assessments are likely reflections of the trust they have in their physicians and the guided journey they have experienced.

### Limitations

This study has limitations. First, next-of-kin^54,55,56^ reports on the beliefs and experiences of decedents are not always accurate. Second, the study did not ascertain the timing on advance care planning activities. However, whenever they took place, they were sufficient to ensure that wishes were largely met. Third, this study population includes only insured individuals in an integrated health care system, and these findings may not be generalizable to patients in other systems.

## Conclusions

Although there is certainly room for improvement in end-of-life care, the scores received from the next of kin of decedents in this study are encouraging. According to the survey respondents, most decedents discussed their end-of-life preferences with next of kin, believed their end-of-life wishes were met, and perceived that their treatments were appropriate. The goal of providing concordant, high-quality, end-of-life care for those 65 years and older seems within reach.

## Notes

Supplement. **eAppendix 1.** Samples, Sample Overlap, Sample Targets, and Additional Sample Descriptions **eAppendix 2.** Details of the Questionnaire Development Process, Focus Groups, Pre-Tests **eAppendix 3.** Questionnaire **eAppendix 4.** Details of the Survey Administration Procedures **eAppendix 5.** Costs Included in Kaiser Permanente Southern California End-of-Life Study **eAppendix 6.** Comparison of Non-Respondents, Respondents, and Weighted Respondents **eAppendix 7.** Regression Model Development Process, Variables, and Predictions **eAppendix 8.** Level of Pain Among All Decedents, Decedents in Hospice, and Decedents in Palliative Care **eAppendix 9.** Percent of Californians, by Age Group, Who Have Written Document on Treatment Wishes, Thought About Wishes for Medical Care at End of Life, Discussed End-of-Life Wishes With a Loved One (from California Health Care Foundation Survey)
