# Supplementary material for: Concordance of End-of-Life Care With End-of-Life Wishes in an Integrated Health Care System
Source: JAMA Netw Open. 2021 Apr 6;4(4):e213053. doi: 10.1001/jamanetworkopen.2021.3053 (PMC8025115; doi:10.1001/jamanetworkopen.2021.3053)
Supplement: Supplement. — eAppendix 1. Samples, Sample Overlap, Sample Targets, and Additional Sample Descriptions eAppendix 2. Details of the Questionnaire Development Process, Focus Groups, Pre-Tests eAppendix 3. Questionnaire eAppendix 4. Details of the Survey Administration Procedures eAppendix 5. Costs Included in Kaiser Permanente Southern California End-of-Life Study eAppendix 6. Comparison of Non-Respondents, Respondents, and Weighted Respondents eAppendix 7. Regression Model Development Process, Variables, and Predictions eAppendix 8. Level of Pain Among All Decedents, Decedents in Hospice, and Decedents in Palliative Care eAppendix 9. Percent of Californians, by Age Group, Who Have Written Document on Treatment Wishes, Thought About Wishes for Medical Care at End of Life, Discussed End-of-Life Wishes With a Loved One (from California Health Care Foundation Survey) [file jamanetwopen-e213053-s001.pdf]

Supplementary Online Content

Glass DP, Wang SE, Minardi PM, Kanter MH. Concordance of end-of-life care with end-of-life wishes in an integrated health care system. *JAMA Netw Open*. 2021;4(4):e213053. doi:10.1001/jamanetworkopen.2021.3053

eAppendix 1. Samples, Sample Overlap, Sample Targets, and Additional Sample Descriptions

eAppendix 2. Details of the Questionnaire Development Process, Focus Groups, Pre-Tests

eAppendix 3. Questionnaire

eAppendix 4. Details of the Survey Administration Procedures

eAppendix 5. Costs Included in Kaiser Permanente Southern California End-of-Life Study

eAppendix 6. Comparison of Non-Respondents, Respondents, and Weighted Respondents

eAppendix 7. Regression Model Development Process, Variables, and Predictions

eAppendix 8. Level of Pain Among All Decedents, Decedents in Hospice, and Decedents in Palliative Care

eAppendix 9. Percent of Californians, by Age Group, Who Have Written Document on Treatment Wishes, Thought About Wishes For Medical Care at End of Life, Discussed End-Of-Life Wishes With a Loved One (From California Health Care Foundation Survey)

This supplementary material has been provided by the authors to give readers additional information about their work.

© 2021 Glass DP et al. *JAMA Network Open.*

eAppendix 1. Samples, Sample overlap, Sample targets, and additional Sample descriptions

Sample Description

This retrospective cross-sectional mortality follow-back survey involved three samples. The first sample was 715 decedents, age 65 or up, who died between April 1 and May 31, 2017. The second was a "high cost" sample - 332 decedents, age 65 or up, who died between June 1, 2016 and May 31, 2017 and whose costs in the last year of life were in the top 10% of the costs of all decedents. The third was a "lower cost" sample with 655 decedents, whose costs were not in the top 10%.

Sample Overlaps

There is an intentional overlap of the samples which was designed to efficiently examine the differences between the "high cost" and "lower cost" decedents. This was implemented with the advice and guidance of Senior Statistician at the Kaiser Permanente Center for Effectiveness & Safety Research.

The "high cost" sample includes 276 decedents sampled from June 2016 through May 2017 whose costs during the last year of life were in the top 10% of the costs of all members who died during this period. We added to this "high cost" sample 56 decedents from the "All Decedent Sample" who were also in this top 10% cost category. We, then, had a total of 332 decedents in the "high cost" sample – who were then appropriately weighted to represent all "high cost" decedents across the year.

The "lower cost" sample are the 655 decedents in the "All Decedent Sample" whose costs were below the 90% percentile of costs in the last year of life. They were weighted to represent all "low cost" decedents across the year.

Sample Targets

We determined our desired sample sizes with the advice and guidance of the Senior Statistician at the Kaiser Permanente Center for Effectiveness & Safety Research.

The desired sample size was determined using statistical power calculations (seeking to exceed 80%).

The All Decedent Sample with 715 respondents has a sample error of ±3.5% which we feel is sufficient precision for our QI purposes and examining the landscape of attitudes about end-of-life care.

We, of course, also wanted to examine differences between the higher cost and lower cost decedents and between those decedents with "do everything" vs "allow to die" attitudes and target samples were selected that we felt would give us sufficient precision for our purposes. The actual samples obtained closely matched with our desired sample sizes.

Additional Sample Descriptions

A. Rationale and Details for 2 visit criteria for inclusion in sample

We opted to select only decedents with 2 or more visits in the last year of life so that there was some experience with our care delivery system and that our questions would make sense. Those with less than 2 visits constituted 2.6% of all those age 65+ who died over a year in our system (0.9% with 1 visit and 1.7% with zero visits). Given these small percentages, there is little obvious downside (or upside) in

© 2021 Glass DP et al. *JAMA Network Open.*

choosing to interview those with 1 visit vs. 2+ visits in the last year of life.

B. Relationships of Respondents to Decedents

The respondents were the primary contact in the decedent's medical record. We asked respondents about their relationship to the decedent in the survey. Here are the results.

**Question 1 First, please tell us how you are related to [Deceased's Name]?**					
		Frequency	Percent	Valid Percent	Cumulative Percent
Valid	Spouse	310	43.4	45.2	45.2
	Parent	34	4.8	5.0	50.2
	Child	264	37.0	38.5	88.7
	Sibling	28	3.9	4.0	92.7
	Other relative	24	3.3	3.5	96.2
	Ex-Spouse	3	.4	.4	96.6
	Friend	5	.7	.8	97.3
	Partner	2	.2	.2	97.5
	Power of Attorney/Legal Guardian/Caretaker	6	.8	.8	98.4
	Other	11	1.5	1.6	100.0
	Total	687	96.1	100.0	
Missing	Multiple mark	26	3.7		
	No response	2	.2		
	Total	28	3.9		
Total		715	100.0		

eAppendix 2. Details of Questionnaire Development Process, Focus Groups, Pre-Tests

We have cited in our footnotes the wide variety of survey questionnaires that we examined as background to drafting our questionnaire (see footnotes 5, 12, 30-38). We did not find an existing instrument that met our goals for examining communications, concordance, treatments, and end-of-life values across a wide variety of settings in our integrated healthcare system. We used existing validated questions from these various surveys where they matched our purposes and drafted original questions when needed. The questionnaire we developed is provided in eAppendix 3 in the Supplement.

Focus Groups

Kaiser Permanente conducted six focus groups in Encino and Los Angeles, California, on June 2, 3, and 4, 2015. Two groups were conducted among Seniors (age 62 to 72) who were currently KP members. Some of these KP members were retired and some were still working. Four groups were with Next-of-Kin (NOK) family members of KP members (aged 60+) who had passed away during the previous 3 to 18 months. Two of the four groups specifically included loved ones who had spent extended amounts of time in the hospital during the last year of their life (a minimum of seven days in the hospital, but most with lengthier stays in the hospital). In the Next-of-Kin groups, the deceased had been a KP member, but the Next-of-Kin may or may not have been a KP member. A mix of ages, employment status, and ethnicities were represented. The groups were comprised of 7 to 9 respondents. A total of 52 respondents attended the six groups. The sessions lasted two hours.

These groups had several objectives, including: 1). to understand the validity and practicality of collecting information from next-of-kin about how well the wishes of a deceased KP member have been met; 2). conducting cognitive tests on some concordance questions and on some scenario questions to help obtain the end-of-life values of decedents. Anyone wishing to obtain the questionnaires used in the focus groups should write to the Corresponding Author for a copy. Questions 4, 6, 15, and 16 in the longer version of the questionnaire contained in eAppendix 3 were among the questions tested in these focus groups.

Key results of the focus groups were that: 1) next-of-kin were very confident they could reflect the wishes of their loved ones (clearly felt they could separate their own values from those of their loved ones) and said they had had end-of-life discussions; 2) that next-of-kin understood and could answer the questions noted above; and, 3) that next-of-kin had a great deal of consensus that the amount of care Kaiser Permanente had given was the "right amount", even in situations when their loved one had experienced extensive hospital stays.

Pre-Test of Survey

We pre-tested the survey in October and November 2017. The survey was mailed to 75 next-of-kin and 6 phone calls were completed (and taped for review). We examined the results of the completed mail surveys, listened to the phone calls, and discussed the questionnaire in a debriefing session with the phone interviewers.

The results from the pre-test included:

- Rewording and simplyfying the scenario questions (questions 15 and 16 above) to better accommodate interviews on the phone;

© 2021 Glass DP et al. *JAMA Network Open.*

- Verifying that respondents felt comfortable and confident that they could answer the questions asked about their loved one’s preferences;
- Dropping a mail post-card request to pass us along to a more familiar family member if this contact was not the family member who was most familiar with the loved one’s situation (we obtained no responses from this request);
- Deciding to create a slightly shorter version of the questionnaire which was fielded with a random 75% of the sample to reduce the length of the interview and the burden on the respondents.

© 2021 Glass DP et al. *JAMA Network Open.*

eAppendix 3. Questionnaire

The survey questionnaire for our study is below. Two slightly different versions of the questionnaire were fielded. Version #1 (the shorter, core version) of the questionnaire is the first one below and has 42 questions. Version #2 (the longer version) of the questionnaire is the second one contained below and has 46 questions. We created the longer version to explore 4 additional questions, however we did not want to burden all the respondents with those test questions.

The shorter version was sent to a random 75% of the sample and the longer version was sent to a random 25% of the sample. The completed interviews reflected this strategy with 76% of the All Decedent sample completing the shorter version of the survey (544 of 715 completes) and 75% of the high cost sample completing the shorter version of the survey (250 of 332 completes).

The additional questions in the longer version of the questionnaire are the questions numbered 4, 5, 14, and 15 in the longer version below (Version #2). Questions 14 and 15 were scenarios that were designed to provide more nuance around the values / beliefs of those on the "do everything" vs "allow to die" question (question 12 in the shorter version of the questionnaire). The results were checked for any question order effects for the two versions of the questionnaire and none were found.

[Go to next page to see Questionnaire]

Version #1: Shorter, Core Version of Questionnaire - page 1 (of 6)

KAISER PERMANENTE®

SURVEY INSTRUCTIONS

- Answer all the questions by checking the box to the left of your answer.
- You are sometimes told to skip over some questions in this survey. When this happens you will see an arrow with a note that tells you what question to answer next, like this:
  ☒ Yes ➔ *Go to Question 1*
  ☐ No

> You are among only a few people we are asking to assist us in improving the quality of Kaiser Permanente's end-of-life care.
> You may choose to answer this survey or not. If you choose not to, this will not affect the care or benefits you get. If you want to know more about this study, please call 1-877-720-8893, ext. 4180.

YOUR LOVED ONE'S CARE AND TREATMENT

1. How are you related to <<First_Name>> <<Last_Name>>?

☐ Spouse
☐ Parent
☐ Child
☐ Sibling
☐ Other relative
☐ Ex-Spouse
☐ Friend
☐ Partner
☐ Power of Attorney/Legal Guardian/ Caretaker
☐ Other

2. **How familiar were you with your loved one's health care decisions in the last year of their life?**

☐ Very familiar
☐ Somewhat familiar
☐ Not too familiar
☐ Not at all familiar
☐ Don't know/not sure

3. **Which of the following three situations best describes the circumstances of your loved one's death?**

☐ "There was a point when the doctors said that they only had a certain amount of time left"
☐ "He or she was sick or injured, but it was not clear that they were going to pass away"
☐ "He or she died from a sudden illness or accident"
☐ Other

4. **Thinking about it <u>from your loved one's perspective</u>, how much do you agree with the following statement:**

"Kaiser Permanente gave care and treatment over the last year of my loved one's life that met my loved one's wishes."

☐ Strongly agree ➔ *Go to Question 6*
☐ Agree ➔ *Go to Question 6*
☐ Disagree
☐ Strongly disagree
☐ Don't know/not sure ➔ *Go to Question 6*

© 2021 Glass DP et al. *JAMA Network Open.*

5. **Why do you disagree?**

____
____
____
____

6. **Had your loved one given real thought to the treatments they wanted or did not want at the end-of-life or had they not thought about it very much?**
   - ☐ Had given real thought to end-of-life treatments
   - ☐ Had not thought much about end-of-life treatments
   - ☐ Don't know/not sure

7. **Did your loved one ever discuss their end-of-life care and treatment preferences with you?**
   - ☐ Yes
   - ☐ No
   - ☐ Don't know/not sure

8. **How much did you know about your loved one's end-of-life care and treatment preferences?**
   - ☐ A lot
   - ☐ Some
   - ☐ Not too much
   - ☐ Nothing at all
   - ☐ Don't know/not sure

9. **Did your loved one have an Advance Directive?**
   - ☐ Yes
   - ☐ No
   - ☐ Don't know/not sure

10. **Did your loved one ever have a discussion with a Kaiser Permanente physician, nurse, or other staff about their end-of-life treatment preferences?**
    - ☐ Yes
    - ☐ No ➔ *Go to Question 12*
    - ☐ Don't know/not sure ➔ *Go to Question 12*

11. **Did a Kaiser Permanente physician, nurse, or other staff ever have an in-depth discussion with your loved one to help them think through his or her care and treatment preferences (for example, discussing the trade-offs your loved one was wanting to make or not make to prolong his or her life)?**
    - ☐ Yes
    - ☐ No
    - ☐ Don't know/not sure

12. **Which of these two statements came closer to the views of your loved one during the last year of their life?**
    - ☐ "In all circumstances, doctors and nurses should do everything possible to save the life of a patient"
    - ☐ "Sometimes there are circumstances where a patient should be allowed to die"
    - ☐ Don't know/not sure

13. **Before your loved one died, did the issue of whether to withhold or stop life-sustaining treatment come up?**
    - ☐ Yes
    - ☐ No
    - ☐ Don't know/not sure

© 2021 Glass DP et al. *JAMA Network Open.*

14. Did your loved one receive from Kaiser Permanente any treatment in the last year of their life that they did not want?
- ☐ Yes
- ☐ No → *Go to Question 16*
- ☐ Don't know/not sure → *Go to Question 16*

15. What was this treatment they did not want?

____
____
____
____

16. Did your loved one not receive from Kaiser Permanente any treatment in the last year of their life that they wanted?
- ☐ Yes
- ☐ No → *Go to Question 18*
- ☐ Don't know/not sure → *Go to Question 18*

17. What was this treatment they wanted?

____
____
____
____

18. Did your loved one receive any of the following treatments in the last year of their life?

	Yes	No	Don't Know / Not Sure
a) Cardiopulmonary resuscitation (CPR) - the use of pressure on the chest, electric shock, and artificial breathing to revive someone	☐	☐	☐
b) Mechanical Respiration - breathing by machine through a tube in the throat	☐	☐	☐
c) Artificial Feeding - giving food and water through a tube inserted into the stomach	☐	☐	☐

19. Did your loved one want to have these treatments, if needed to keep him/her alive, during the last year of their life?

	Yes	No	Don't Know / Not Sure
a) Cardiopulmonary resuscitation (CPR) - the use of pressure on the chest, electric shock, and artificial breathing to revive someone	☐	☐	☐
b) Mechanical Respiration - breathing by machine through a tube in the throat	☐	☐	☐
c) Artificial Feeding - giving food and water through a tube inserted into the stomach	☐	☐	☐

© 2021 Glass DP et al. *JAMA Network Open.*

20. **Where did your loved one die?**
    - ☐ In the hospital
    - ☐ At home (or a relative's home)
    - ☐ In a nursing home or skilled nursing facility
    - ☐ In a hospice facility
    - ☐ In an assisted living facility
    - ☐ Somewhere else
    - ☐ Don't know/not sure

21. **Approximately how many days did your loved one spend in the hospital in the last year of their life?**

    __________ Number of days
    - ☐ Don't know/not sure

22. **Do you think your loved one would say the amount of medical care they received from Kaiser Permanente during the last year of their life was too little, too much, or the right amount?**
    - ☐ Too little
    - ☐ Too much
    - ☐ Right amount
    - ☐ Don't know/not sure

23. **In the last month of life, did your loved one have pain or take medicine for pain?**
    - ☐ Yes
    - ☐ No
    - ☐ Don't know/not sure

24. **How often did your loved one's pain make him/her uncomfortable?**
    - ☐ Always
    - ☐ Usually
    - ☐ Sometimes
    - ☐ Never
    - ☐ He/she did not have any pain
    - ☐ Don't know/not sure

25. **Did the Kaiser Permanente doctors and nurses do everything they could to ease your loved one's pain, or could they have done more?**
    - ☐ Did everything they could
    - ☐ Could have done more
    - ☐ Don't know/not sure

26. **Was your loved one receiving hospice care services around the time of their death?**
    - ☐ Yes
    - ☐ No
    - ☐ Don't know/not sure

27. **Apart from hospice care services, did your loved one receive palliative care from Kaiser Permanente?**
    - ☐ Yes
    - ☐ No
    - ☐ Don't know/not sure

28. **Overall, how would you rate the care that your loved one received in the last month of his/her life?**
    - ☐ Excellent
    - ☐ Very good
    - ☐ Good
    - ☐ Fair
    - ☐ Poor
    - ☐ Did not receive any care in last month of life
    - ☐ Don't know/not sure

© 2021 Glass DP et al. *JAMA Network Open.*

ABOUT YOUR LOVED ONE

29. Where did your loved one live during most of the last year of their life?
- ☐ In the hospital
- ☐ At home (or a relative's home)
- ☐ In a nursing home or skilled nursing facility
- ☐ In a hospice facility
- ☐ In an assisted living facility
- ☐ Somewhere else
- ☐ Don't know/not sure

30. As far as you know, where do you think your loved one would have wanted to die if given the choice?
- ☐ In the hospital
- ☐ At home (or a relative's home)
- ☐ In a nursing home or skilled nursing facility
- ☐ In a hospice facility
- ☐ In an assisted living facility
- ☐ Somewhere else
- ☐ Don't know/not sure

31. How important was it to your loved one to die at this desired location?
- ☐ Very important
- ☐ Somewhat important
- ☐ Not too important
- ☐ Not at all important
- ☐ Don't know/not sure

32. How much do you think the doctors and staff tried to accommodate your loved one's desired place to die?
- ☐ A great deal
- ☐ Somewhat
- ☐ Not too much
- ☐ Not at all
- ☐ Don't know/not sure

33. What is the highest grade of education your loved one completed?
- ☐ High school or lower
- ☐ Some college (AA or AS degree)
- ☐ 4-year College or University (BA or BS degree)
- ☐ Graduate or professional school (MA, MS, Ph.D or equivalent)
- ☐ Don't know/not sure

34. What is your best estimate of your loved one's household's total annual income from all sources before taxes in the last year of their life?
- ☐ $20,000 or less
- ☐ $21,000 to $35,000
- ☐ $36,000 to $50,000
- ☐ $51,000 to $70,000
- ☐ $71,000 to $100,000
- ☐ More than $100,000
- ☐ Don't know/not sure

35. Was your loved one Latino or Hispanic?
- ☐ Yes
- ☐ No
- ☐ Don't know/not sure

36. What was your loved one's race?
- ☐ White
- ☐ Asian
- ☐ Black or African American
- ☐ American Indian / Alaska Native
- ☐ Hawaiian / Other Pacific Islander
- ☐ Other
- ☐ Prefer not to say

© 2021 Glass DP et al. *JAMA Network Open.*

37. During the last month of your loved one's life, was your loved one able to make their own health care decisions, or did someone else need to make those decisions for them?
- ☐ Loved one able to make health care decisions
- ☐ Someone else needed to make health care decisions for loved one
- ☐ Don't know/not sure

38. How important was religion in your loved one's life?
- ☐ Very important
- ☐ Somewhat important
- ☐ Not too important
- ☐ Not at all important
- ☐ Don't know/prefer not to say

39. Which religion did your loved one belong to?
- ☐ Protestant (include Baptist, Lutheran, Methodist, Presbyterian, Episcopalian, Pentecostal, Jehovah's Witness, Church of Christ, etc.)
- ☐ Roman Catholic
- ☐ Jewish
- ☐ Mormon (include Church of Jesus Christ of Latter Day Saints)
- ☐ Orthodox (Greek or Russian)
- ☐ Islam/Muslim
- ☐ Other religion
- ☐ No religion, not a believer, atheist, agnostic
- ☐ Don't know/prefer not to say

40. What was your loved one's marital status?
- ☐ Single, never married
- ☐ Married or domestic partner
- ☐ Widowed
- ☐ Divorced
- ☐ Separated
- ☐ Don't know/prefer not to say

ABOUT YOU

41. Are you now or have you been a Kaiser Permanente member?
- ☐ Now a Kaiser Permanente member
- ☐ Have been a Kaiser Permanente member in the past
- ☐ Never been a Kaiser Permanente member

ADDITIONAL COMMENTS

42. Is there anything else you'd like to share about how the care at Kaiser Permanente could have been improved for your loved one?

____
____
____
____
____
____
____
____
____
____

Please print your first and last name below. This is to let us know that you have returned your survey.

First Name: ____________________

Last Name: ____________________

Thank You
Please return the completed survey
in the postage-paid envelope or send to:
DSS Research • P.O. Box 985009
Ft. Worth, TX 76185-5009

© 2021 Glass DP et al. *JAMA Network Open.*

Version #2: Longer Version of Questionnaire - page 1 (of 7)

KAISER PERMANENTE®

SURVEY INSTRUCTIONS

- Answer all the questions by checking the box to the left of your answer.
- You are sometimes told to skip over some questions in this survey. When this happens you will see an arrow with a note that tells you what question to answer next, like this:
  - ☒ Yes ➔ *Go to Question 1*
  - ☐ No

You are among only a few people we are asking to assist us in improving the quality of Kaiser Permanente's end-of-life care.

You may choose to answer this survey or not. If you choose not to, this will not affect the care or benefits you get. If you want to know more about this study, please call 1-877-720-8893, ext. 4180.

YOUR LOVED ONE'S CARE AND TREATMENT

1. How are you related to <<First_Name>> <<Last_Name>>?
   - ☐ Spouse
   - ☐ Parent
   - ☐ Child
   - ☐ Sibling
   - ☐ Other relative
   - ☐ Ex-Spouse
   - ☐ Friend
   - ☐ Partner
   - ☐ Power of Attorney/Legal Guardian/ Caretaker
   - ☐ Other

2. **How familiar were you with your loved one's health care decisions in the last year of their life?**
   - ☐ Very familiar
   - ☐ Somewhat familiar
   - ☐ Not too familiar
   - ☐ Not at all familiar
   - ☐ Don't know/not sure

3. **Which of the following three situations best describes the circumstances of your loved one's death?**
   - ☐ "There was a point when the doctors said that they only had a certain amount of time left"
   - ☐ "He or she was sick or injured, but it was not clear that they were going to pass away"
   - ☐ "He or she died from a sudden illness or accident"
   - ☐ Other

4. **Thinking about it from <u>your loved one's perspective</u>, how satisfied are you that your loved one died in the way that he / she would have wanted to?**
   - ☐ Very Satisfied
   - ☐ Satisfied
   - ☐ Dissatisfied
   - ☐ Very Dissatisfied
   - ☐ Don't know/not sure ➔ *Go to Question 6*

© 2021 Glass DP et al. *JAMA Network Open.*

5. Why do you think your loved one would feel this way?

____
____
____
____
____
____

6. Thinking about it <u>from your loved one's perspective</u>, how much do you agree with the following statement:

"Kaiser Permanente gave care and treatment over the last year of my loved one's life that met my loved one's wishes."

- ☐ Strongly Agree ➔ *Go to Question 8*
- ☐ Agree ➔ *Go to Question 8*
- ☐ Disagree
- ☐ Strongly Disagree
- ☐ Don't know/not sure ➔ *Go to Question 8*

7. Why do you disagree?

____
____
____
____

8. Had your loved one given real thought to the treatments they wanted or did not want at the end-of-life or had they not thought about it very much?

- ☐ Had given real thought to end-of-life treatments
- ☐ Had not thought much about end-of-life treatments
- ☐ Don't know/not sure

9. Did your loved one ever discuss their end-of-life care and treatment preferences with you?

- ☐ Yes
- ☐ No
- ☐ Don't know/not sure

10. How much did you know about your loved one's end-of-life care and treatment preferences?

- ☐ A lot
- ☐ Some
- ☐ Not too much
- ☐ Nothing at all
- ☐ Don't know/not sure

11. Did your loved one have an Advance Directive?

- ☐ Yes
- ☐ No
- ☐ Don't know/not sure

12. Did your loved one ever have a discussion with a Kaiser Permanente physician, nurse, or other staff about their end-of-life treatment preferences?

- ☐ Yes
- ☐ No ➔ *Go to Question 14*
- ☐ Don't know/not sure ➔ *Go to Question 14*

13. Did a Kaiser Permanente physician, nurse, or other staff ever have an in-depth discussion with your loved one to help them think through his or her care and treatment preferences (for example, discussing the trade-offs your loved one was wanting to make or not make to prolong his or her life)?

- ☐ Yes
- ☐ No
- ☐ Don't know/not sure

© 2021 Glass DP et al. *JAMA Network Open.*

14. **Which of these two statements came closer to the views of your loved one during the last year of their life?**
    - ☐ "In all circumstances, doctors and nurses should do everything possible to save the life of a patient"
    - ☐ "Sometimes there are circumstances where a patient should be allowed to die"
    - ☐ Don't know/not sure

We would like to understand your loved one's values in a little more depth. Please indicate what your loved one would have preferred for himself/ herself in the following two situations if they had occurred six months before he/she died:

15. **Situation #1: Your loved one has a sudden accident or stroke.**

    Doctors have determined that your loved one has a brain injury, leaving your loved one unable to recognize himself/herself or their family. The doctors say your loved one is not expected to recover these abilities.

    Would your loved one:
    - ☐ Have wanted life-sustaining treatments to continue as long as possible
    - ☐ Have wanted to stop life-sustaining treatment
    - ☐ Don't know/not sure what loved one would have wanted

16. **Situation #2: Doctors have determined that your loved one has a disease with no hope of improvement, and that illness makes it very hard for your loved one to physically function. Your loved one is bedbound, cannot get out bed to go to the bathroom by themself, needs help in all their daily activities, but has their mental abilities.**

    Would your loved one:
    - ☐ Have wanted life-sustaining treatments to continue as long as possible
    - ☐ Have wanted to stop life-sustaining treatment
    - ☐ Don't know/not sure what loved one would have wanted

17. **Before your loved one died, did the issue of whether to withhold or stop life-sustaining treatment come up?**
    - ☐ Yes
    - ☐ No
    - ☐ Don't know/not sure

18. **Did your loved one receive from Kaiser Permanente any treatment in the last year of their life that they did not want?**
    - ☐ Yes
    - ☐ No ➔ *Go to Question 20*
    - ☐ Don't know/not sure ➔ *Go to Question 20*

19. **What was this treatment they did not want?**

    ____

    ____

    ____

    ____

© 2021 Glass DP et al. *JAMA Network Open.*

20. Did your loved one not receive from Kaiser Permanente any treatment in the last year of their life that they wanted?
    - ☐ Yes
    - ☐ No ➔ *Go to Question 22*
    - ☐ Don't know/not sure ➔ *Go to Question 22*

21. What was this treatment they wanted?

    ____
    ____
    ____
    ____

22. Did your loved one receive any of the following treatments in the last year of their life?

	Yes	No	Don't Know / Not Sure
a) Cardiopulmonary resuscitation (CPR) - the use of pressure on the chest, electric shock, and artificial breathing to revive someone	☐	☐	☐
b) Mechanical Respiration - breathing by machine through a tube in the throat	☐	☐	☐
c) Artificial Feeding - giving food and water through a tube inserted into the stomach	☐	☐	☐

23. Did your loved one want to have these treatments, if needed to keep him/her alive, during the last year of their life?

	Yes	No	Don't Know / Not Sure
a) Cardiopulmonary resuscitation (CPR) - the use of pressure on the chest, electric shock, and artificial breathing to revive someone	☐	☐	☐
b) Mechanical Respiration - breathing by machine through a tube in the throat	☐	☐	☐
c) Artificial Feeding - giving food and water through a tube inserted into the stomach	☐	☐	☐

24. Where did your loved one die?
    - ☐ In the hospital
    - ☐ At home (or a relative's home)
    - ☐ In a nursing home or skilled nursing facility
    - ☐ In a hospice facility
    - ☐ In an assisted living facility
    - ☐ Somewhere else
    - ☐ Don't know/not sure

25. Approximately how many days did your loved one spend in the hospital in the last year of their life?

    __________ Number of days
    - ☐ Don't know/not sure

© 2021 Glass DP et al. *JAMA Network Open.*

26. Do you think your loved one would say the amount of medical care they received from Kaiser Permanente during the last year of their life was too little, too much, or the right amount?

- ☐ Too little
- ☐ Too much
- ☐ Right amount
- ☐ Don't know/not sure

27. In the last month of life, did your loved one have pain or take medicine for pain?

- ☐ Yes
- ☐ No
- ☐ Don't know/not sure

28. How often did your loved one's pain make him/her uncomfortable?

- ☐ Always
- ☐ Usually
- ☐ Sometimes
- ☐ Never
- ☐ He/She did not have any pain
- ☐ Don't know/not sure

29. Did the Kaiser Permanente doctors and nurses do everything they could to ease your loved one's pain, or could they have done more?

- ☐ Did everything they could
- ☐ Could have done more
- ☐ Don't know/not sure

30. Was your loved one receiving hospice care services around the time of their death?

- ☐ Yes
- ☐ No
- ☐ Don't know/not sure

31. Apart from hospice care services, did your loved one receive palliative care from Kaiser Permanente?

- ☐ Yes
- ☐ No
- ☐ Don't know/not sure

32. Overall, how would you rate the care that your loved one received in the last month of his/her life?

- ☐ Excellent
- ☐ Very good
- ☐ Good
- ☐ Fair
- ☐ Poor
- ☐ Did not receive any care in last month of life
- ☐ Don't know/not sure

ABOUT YOUR LOVED ONE

33. Where did your loved one live during most of the last year of their life?

- ☐ In the hospital
- ☐ At home (or a relative's home)
- ☐ In a nursing home or skilled nursing facility
- ☐ In a hospice facility
- ☐ In an assisted living facility
- ☐ Somewhere else
- ☐ Don't know/not sure

34. As far as you know, where do you think your loved one would have wanted to die if given the choice?

- ☐ In the hospital
- ☐ At home (or a relative's home)
- ☐ In a nursing home or skilled nursing facility
- ☐ In a hospice facility
- ☐ In an assisted living facility
- ☐ Somewhere else
- ☐ Don't know/not sure

© 2021 Glass DP et al. *JAMA Network Open.*

35. **How important was it to your loved one to die at this desired location?**
- ☐ Very important
- ☐ Somewhat important
- ☐ Not too important
- ☐ Not at all important
- ☐ Don't know/not sure

36. **How much do you think the doctors and staff tried to accommodate your loved one's desired place to die?**
- ☐ A great deal
- ☐ Somewhat
- ☐ Not too much
- ☐ Not at all
- ☐ Don't know/not sure

37. **What is the highest grade of education your loved one completed?**
- ☐ High school or lower
- ☐ Some college (AA or AS degree)
- ☐ 4-year College or University (BA or BS degree)
- ☐ Graduate or professional school (MA, MS, Ph.D or equivalent)
- ☐ Don't know/not sure

38. **What is your best estimate of your loved one's household's total annual income from all sources before taxes in the last year of their life?**
- ☐ $20,000 or less
- ☐ $21,000 to $35,000
- ☐ $36,000 to $50,000
- ☐ $51,000 to $70,000
- ☐ $71,000 to $100,000
- ☐ More than $100,000
- ☐ Don't know/not sure

39. **Was your loved one Latino or Hispanic?**
- ☐ Yes
- ☐ No
- ☐ Don't know/not sure

40. **What was your loved one's race?**
- ☐ White
- ☐ Asian
- ☐ Black or African American
- ☐ American Indian / Alaska Native
- ☐ Hawaiian / Other Pacific Islander
- ☐ Other
- ☐ Prefer not to say

41. **During the last month of your loved one's life, was your loved one able to make their own health care decisions, or did someone else need to make those decisions for them?**
- ☐ Loved one able to make health care decisions
- ☐ Someone else needed to make health care decisions for loved one
- ☐ Don't know/not sure

42. **How important was religion in your loved one's life?**
- ☐ Very important
- ☐ Somewhat important
- ☐ Not too important
- ☐ Not at all important
- ☐ Don't know/prefer not to say

© 2021 Glass DP et al. *JAMA Network Open.*

43. **Which religion did your loved one belong to?**
    - ☐ Protestant (include Baptist, Lutheran, Methodist, Presbyterian, Episcopalian, Pentecostal, Jehovah's Witness, Church of Christ, etc.)
    - ☐ Roman Catholic
    - ☐ Jewish
    - ☐ Mormon (include Church of Jesus Christ of Latter Day Saints)
    - ☐ Orthodox (Greek or Russian)
    - ☐ Islam/Muslim
    - ☐ Other religion
    - ☐ No religion, not a believer, atheist, agnostic
    - ☐ Don't know/prefer not to say

44. **What was your loved one's marital status?**
    - ☐ Single, never married
    - ☐ Married or domestic partner
    - ☐ Widowed
    - ☐ Divorced
    - ☐ Separated
    - ☐ Don't know/prefer not to say

ABOUT YOU

45. **Are you now or have you been a Kaiser Permanente member?**
    - ☐ Now a Kaiser Permanente member
    - ☐ Have been a Kaiser Permanente member in the past
    - ☐ Never been a Kaiser Permanente member

ADDITIONAL COMMENTS

46. **Is there anything else you'd like to share about how the care at Kaiser Permanente could have been improved for your loved one?**

____
____
____
____
____
____
____
____
____
____

Please print your first and last name below. This is to let us know that you have returned your survey.

First Name: ______________________________

Last Name: ______________________________

Thank You
Please return the completed survey in the postage-paid envelope or send to:
DSS Research • P.O. Box 985009
Ft. Worth, TX 76185-5009

© 2021 Glass DP et al. *JAMA Network Open.*

eAppendix 4. Details of Survey Administration Procedures

The details of the administration of this survey might be of interest to others involved in the design or administration of other end-of-life surveys. Here are some of the details about our survey. Interested parties are encouraged to contact the corresponding author if there are other aspects about the survey administration or procedures not covered here that they would like to understand.

Time Frame for Fielding the Survey

The time frame was chosen for the All Decedent Sample to allow the next-of-kin interviews to take place approximately 6 months after the death of the decedent. In focus groups, next-of-kin indicated that this was the appropriate time to interview them on end-of-life issues following the passing of their loved one. The interviews with next-of-kin were conducted between December 19 2017 to February 7 2018 for decedents who had passed in April and May of 2017.

Respondent Identification

The respondent was the person listed as the primary contact in the decedent’s electronic medical record.

Relationship of Respondents to the Decedents

The respondents were overwhelmingly bereaved family members related to the decedent. A table showing the details of those relationships is contained in eAppendix 1.

Voluntary Participation in the Survey

Any potential respondent for the survey was informed both in the cover letter (or in the introduction on the phone) and on the first page of the questionnaire that participation in the survey was voluntary. Potential participants were offered clear opportunities to decline to participate prior to starting the survey. This wording was reviewed by the IRB.

Mode of conducting the survey

Two mailings of the questionnaire were sent to the address of the primary contact if we had it (about 35% of the time) or otherwise to the decedent’s address. If no response was received, up to a maximum of 10 phone calls were made to next-of-kin. However, 85% of the completions were by mail (involving no phone calls) and 15% were completed by phone. The mean number of contacts was 2.1 per respondent.

The determination to go to a maximum 10 call protocol was made with the advice of the survey vendor after receiving a low response rate in the pre-test of the survey. The maximum 10 call protocol does not mean most respondents received 10 calls (as just noted above). A message was only left only on the 6th phone attempt. There was no message left on any other phone attempt.

Handling of Distressed Respondents

Phone interviewers were provided with protocols and briefed on how to handle to any respondents exhibiting signs of distress or grief. Phone interviewers were provided with a reference phone number to grief counselors for any respondent that seemed to need or want that kind of support. The

© 2021 Glass DP et al. *JAMA Network Open.*

interviewers were also instructed to notify managers of any distressed respondents. There were no occurrences of this during the fielding of the survey.

© 2021 Glass DP et al. *JAMA Network Open.*

eAppendix 5. Costs included in Kaiser Permanente Southern California End-of-Life Study

Source: Kaiser Permanente Health Plan Accounting

Kaiser Permanente derives cost data for patients and encounters using a systematic cost accounting methodology (for within-system utilization) and paid claims data (for outside-system utilization). The categories are inclusive of all services used by Kaiser Permanente members. The costs we are using here are those used to guide our business and operational decisions.

	Kaiser Permanente (Inside Medical)	Outside Medical (OM)
Hospital	Yes	Yes
Home Health	Yes	Yes
Hospice	Yes	Yes
SNF	No	Yes
Clinic	Yes	Yes
Outpatient Pharmacy	Yes	No
Outpatient Lab	Yes	No
Outpatient Radiology	Yes	No
Home Health Pharmacy	Yes	No
Ambulance	No	Yes
Dialysis	No	Yes

© 2021 Glass DP et al. *JAMA Network Open.*

eAppendix 6. Comparison of Non-Respondents, Respondents, and Weighted Respondents*

Non-response weights were developed using logistic regression. As shown in the table below, the included predictors were age, race, gender, and costs during the last year of life.

Characteristics	Non-Respondents (n=1566)	Unweighted Respondents (n=715)	P Value	Total = Non-Resp. + Resp. (N=2281)	Weighted Respondents (n=715)
Age, n(%)			.009		
65-74 years old	484 (31%)	181 (25%)		665 (29%)	207 (29%)
75-84 years old	521 (33%)	229 (32%)		750 (33%)	239 (33%)
85-94 years old	471 (30%)	254 (36%)		725 (32%)	229 (32%)
95-108 years old	90 (6%)	51 (7%)		141 (6%)	41 (6%)
Race, n(%)			<.001		
White	856 (55%)	502 (71%)		1358 (60%)	431 (60%)
Black	249 (16%)	66 (9%)		315 (14%)	98 (14%)
Latino	295 (19%)	92 (13%)		387 (17%)	122 (17%)
Asian	118 (8%)	38 (5%)		156 (7%)	49 (7%)
Other-Unknown	32 (2%)	9 (1%)		41 (2%)	15 (2%)
Gender, n(%)			.003		
Female	812 (52%)	323 (45%)		1135 (50%)	354 (50%)
Male	754 (48%)	392 (55%)		1146 (50%)	361 (51%)
Cost, n(%)			.035		
Bottom 90%	1399 (89%)	659 (92%)		2058 (90%)	655 (92%)
Top 10%	167 (11%)	56 (8%)		223 (10%)	60 (8%)
*All Decedent Sample					

© 2021 Glass DP et al. *JAMA Network Open.*

eAppendix 7. Regression Model Development Process, Variables, and Predictions

Regression Development and Results

The steps we employed in developing our regression model included the following.

Our dependent variable was our overall concordance variable ("Kaiser Permanente gave care and treatment over the last year of my loved one's life that met my loved one's wishes.").

We identified variables that we hypothesized a priori might be associated with concordance based on the literature. These variables were measures that fit into the three key areas that both theoretically and in the literature seemed likely to be associated with concordance: 1. End-of-life preferences and attitudes (broad values, preferences around specific treatments, etc.); 2. Communications (Discussions with medical staff and/or next-of-kin, existence of Advance Directive); 3. Actions of / Experiences with Kaiser Permanente (treatments given, pain management, amount of care, money spent in last year, accommodations around desired location of death, etc.). We also included decedents' demographic information.

We conducted bivariate analyses to determine which variables were significantly associated with concordance. This included 2-tailed t tests for continuous variables and Pearson Chi Square tests for categorical variables. We used the results of these analyses to inform our Ordinary Least Squares regression analyses. We tested and reviewed alternative models, examining the relative impact of various measures, winnowing out those variables that were not significant (significance was defined as ≤ .05), to arrive at a final model.

The final Ordinary Least Squares (OLS) regression model included the following 6 variables:

1. MD or Staff Discussions about End-of-Life Preferences
2. Receiving Treatments that were not wanted
3. Not Receiving Treatments wanted
4. MD could have done more for those who were in pain
5. MDs or staff tried to accommodate loved one's desired place to die
6. Received too little care in last year of life

The R square on this Ordinary Least Square regression was .43.

Regression Predictions

The OLS regression equation, from the analyses described above, was then used to produce predicted scores for the dependent variable ("Kaiser Permanente gave care and treatment over the last year of my loved one's life that met my loved one's wishes."). We then changed each independent variable, one at

© 2021 Glass DP et al. *JAMA Network Open.*

a time, raising all scores on that independent variable to the maximum ("best") score and examined the predicted changed on the dependent variable.

For example, on Table 2, we show that 65.1% of decedents had a discussion with physicians or staff about end-of-life treatment preferences (55.4% had an in-depth discussion and 9.7% had a regular discussion). We changed that variable so that 100% of decedents had had a discussion with physicians or staff about end-of-life treatment preferences and reran the equation to produce a prediction of the change in independent variable. The resulting predicted percentage point change on those reporting they "strongly agreed" that Kaiser had met their loved one's wishes was an increase of 9.8 percentage points. This is what is shown in Figure 1.

A description of this approach can be found in Graubard, B., and E. Korn. 1999. "Predictive Margins with Survey Data." Biometrics 55 (2): 652–9.

© 2021 Glass DP et al. *JAMA Network Open.*

eAppendix 8. Level of Pain among All Decedents, Decedents in Hospice, and Decedents in Palliative Care*

Level of Pain among All Decedents, Decedents in Hospice, and Decedents in Palliative Care*			
**Measure**	**All Decedents**	**Decedents in Hospice**	**Decedents in Palliative Care**
	**N (%)**	**N (%)**	**N (%)**
**How often loved one's pain made them uncomfortable:**			
Always	182 (28.4)	96 (26.0)	70 (27.7)
Usually	147 (22.8)	91(24.7)	61 (24.1)
Sometimes	219 (34.1)	133 (36.0)	88 (34.8)
Never / Did not have any pain	94 (14.7)	49 (13.3)	34 (13.5)
*Source: All Decedent Sample.			

© 2021 Glass DP et al. *JAMA Network Open.*

eAppendix 9. Percent of Californians, by age group, who have written document on treatment wishes, thought about wishes for medical care at end of life, discussed end-of-life wishes with a loved one (from California Health Care Foundation Survey).

eAppendix 9 - Figure 1.
Percent of Californians who have a written document regarding end-of-life medical treatment wishes by Age

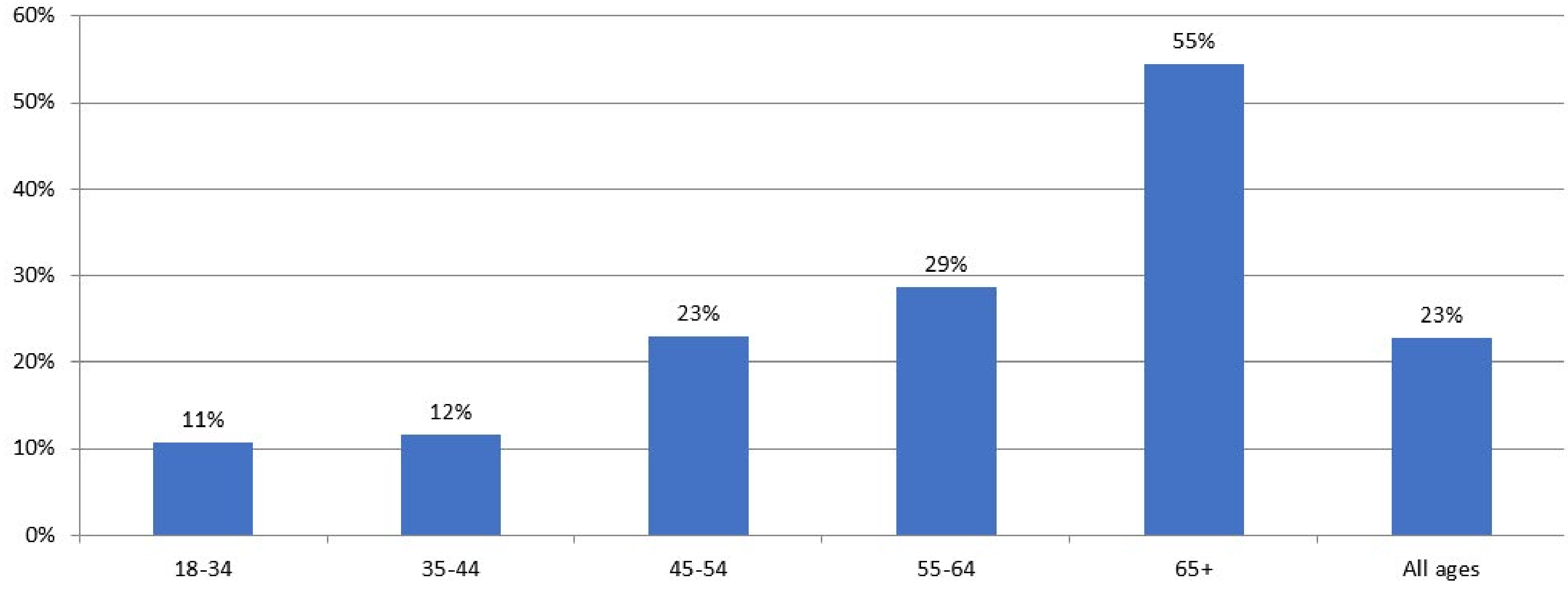

* Source: California Health Care Foundation: Final Chapter: Californians' Attitudes and Experiences with Death and Dying, 2011. Base: N = 1,669.

© 2021 Glass DP et al. *JAMA Network Open.*

eAppendix 9 - Figure 2.
Percent of Californians who have thought about wishes for medical care at end of life by Age
(% "a great deal" or "some")

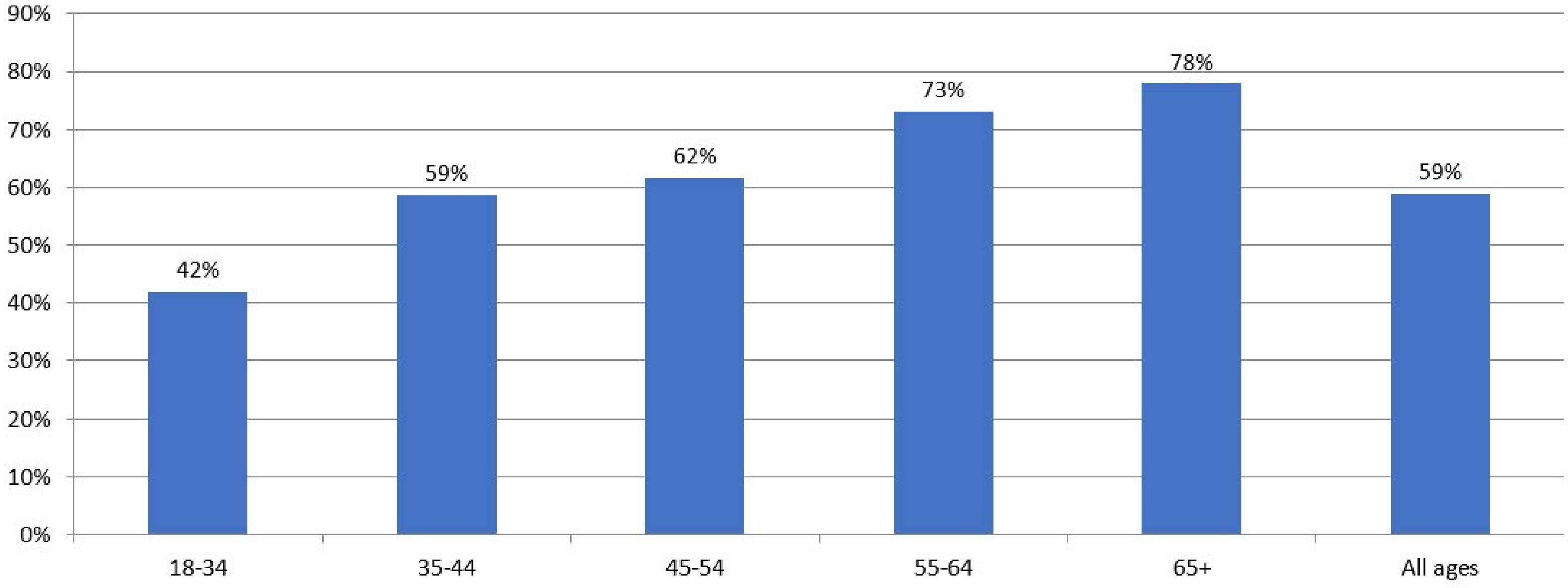

* Source: California Health Care Foundation: Final Chapter: Californians' Attitudes and Experiences with Death and Dying, 2011. Base: N = 1,669.

© 2021 Glass DP et al. *JAMA Network Open.*

eAppendix 9 - Figure 3.
Percent of Californians who have discussed end-of-life wishes with a loved one by Age

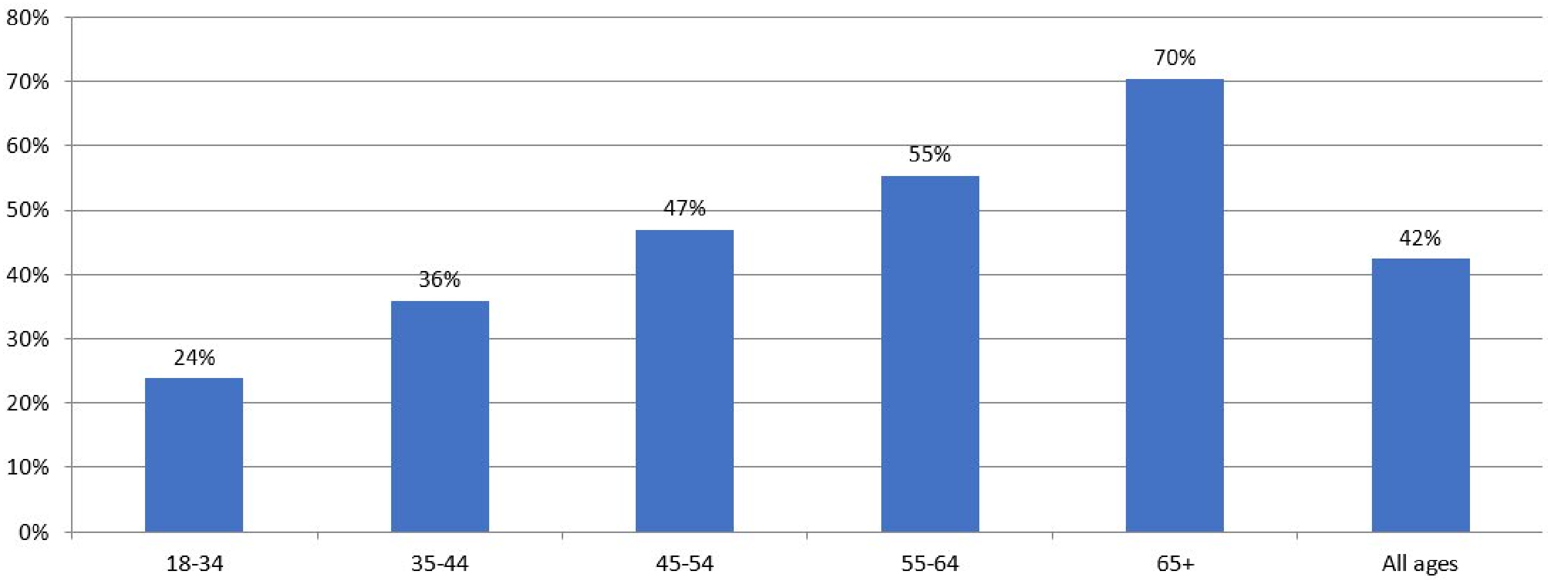

* Source: California Health Care Foundation: Final Chapter: Californians' Attitudes and Experiences with Death and Dying, 2011. Base: N = 1,669.

© 2021 Glass DP et al. *JAMA Network Open.*
